# The European MR safety landscape

**DOI:** 10.1186/s13244-024-01813-6

**Published:** 2024-10-07

**Authors:** European Society of Radiology (ESR), Francesco Santini, Anna Pichiecchio, Megan McFadden, Núria Bargalló, Emanuele Neri, Anne Dorte Blankholm, Simone Busoni, Siegfried Trattnig

**Affiliations:** European Society of Radiology, Vienna, Austria

**Keywords:** MR safety, Education, Guidelines, Accidents, Implants

## Abstract

**Objectives:**

Despite the absence of ionizing radiation, magnetic resonance (MR) has inherent risks in clinical practice that can have serious health consequences if overlooked. At an international level, there are MR safety guidelines that help define the organization of a radiology department to minimize the risks for patients and personnel. However, competing guidelines exist and not every country and institution adheres to the same standards. In this work, we aim to understand the current situation regarding MR safety practices across Europe, and to identify the points where harmonization, coordination, or further education is needed.

**Methods:**

An anonymous survey questionnaire was distributed between April and June 2023 through ESR member societies to healthcare professionals, aimed to assess personnel training, local policies, scanning practices, and accidents.

**Results:**

Seven hundred and ninety-three responses were obtained from 44 different countries. The majority of respondents from five countries reported that MR safety is mandated by law, but we could only confirm two (Italy and Austria). While 77% of the responses said that their institution had a clear MR safety guideline, 52% said that nobody in their institution had received specific MR safety training. MR-conditional cardiac devices are mostly scanned in university hospitals (reported by 75% of respondents from this type of institution) but in only 42% of outpatient facilities. MR-unsafe cardiac devices are only scanned off-label in 27% of university hospitals, and in an even smaller share of other institutions. Approximately 12% of the respondents reported MR-related accidents resulting in patient or personnel injury. Overall, there is the sentiment that MR safety education and regulation are needed.

**Conclusions:**

The European landscape in terms of MR safety is very heterogeneous, with different regulations across countries, and different procedures for MR safety training and their application in clinical routine. The European Society of Radiology is optimally positioned to play an active role in the harmonization of MR safety education and practices across Europe, and we are proposing a four-tiered framework for the development of a teaching curriculum for MR safety training.

**Critical relevance statement:**

There is room for raising awareness of MR safety issues to ensure patient safety, reduce accidents, and benefit more patients. We advocate for radiologist-led standardization and improvement of MR safety training as a way to address this problem.

**Key Points:**

- Our survey of MR safety practices across Europe revealed significant heterogeneity in regulations, training, and scanning practices.
- There is a widespread lack of awareness and implementation of MR safety guidelines and diffuse uncertainty, under-scanning of eligible patients, and preventable accidents.
- The ESR proposes a harmonized, four-tiered MR safety training curriculum to standardize, and improve safety practices across Europe.

## Introduction

Magnetic resonance (MR) imaging is the method of choice for imaging the human body without using ionizing radiation and offers at the same time high resolution morphological, metabolic, and functional images. While commonly regarded as a “safer” alternative to techniques based on ionizing radiation, MR uses three different fields that pose significant security risks in themselves, potentially resulting in fatalities due to their interaction with magnetic and conductive materials.

The greatest risk associated with MR is posed by the static magnetic field, which, for most clinical scanners, is always active. When a ferromagnetic object is introduced in the vicinity of the scanner, an attractive force of up to several hundred kilograms might act upon it, depending on the volume of the object itself. Everyday medical objects, such as chairs, beds, or oxygen flasks, can all result in fatal injuries when a person is hit by these flying objects or caught between them and the magnet [1].

Gradient fields, and, even more relevantly, radiofrequency fields are active during the acquisition, and in themselves pose significant safety concerns, such as heating, focal burns, and acoustic noise [2]. Patients with active and passive implants are especially at risk, due to the potential interaction of the implant with one or more of the magnetic fields of an MR scanner. Furthermore, MR examinations performed under anesthesia and MR-guided interventional procedures imply a significant risk to safety for both patients and staff [3].

Despite the fact that the risks are known, and that safety guidelines are available, it is also evident that the problem of MR-related accidents is real and underreported [1, 4–6]. Within the general framework of the engineering norms [7] and guidelines [8–10] regulating medical devices, which limit the direct exposure of persons to potentially harmful electromagnetic fields, the numerous interactions between MR components and the surrounding environment require specific workflows and procedures. To ensure safety and manage the dangers of an MR suite to patients, staff, and visitors, several professional groups are involved, including radiologists, radiographers, physicists, and engineers.

Concerning the organization of responsibilities, there are at least two leading guidelines existing in the current practice. The first guideline, spearheaded by institutions in the United States of America, but also co-signed by the European Society of Radiology (ESR), the European Society of Magnetic Resonance in Medicine and Biology (ESMRMB), the European Federation of Organizations for Medical Physics (EFOMP), and the European Federation of Radiographer Societies (EFRS) [11, 12], identifies three main actors in the enactment of MR safety: MR medical director (MRMD, usually a radiologist or physician), MR safety officer (MRSO, usually a radiographer or MR technologist), and MR safety expert (MRSE, usually a physicist or engineer). In this structure, the ultimate legal responsibility lies with the MRMD, and a physician should always be available during the scan of the patients. The second guideline, recommended by the EFOMP [13, 14] and reflected in the national guidelines of the United Kingdom [15], doesn’t address the role of MRMD but defines the roles of MRSO (termed MR responsible person in the UK guideline), responsible for MR safety on a day by day basis, who may be carried out by suitably qualified personnel having recorded training (such as medical physicists, radiographers, radiologists, etc.), and of the MRSE.

In either guideline, it is clear that there are always inherent risks associated with MR procedures, and the decision on whether and how to perform a particular scan is not binary and needs to be taken in light of a risk/benefit evaluation. Alongside the radiographer, who performs duties related to the safety, comfort, and satisfaction of the patient, a key role is played by the radiologist, who is ultimately responsible for providing the diagnostic service to the patient and for their wellbeing and is involved in all phases of the radiological examination and physicist who provides physical agents risk assessment and determination of the conditionality status of devices. Radiologists in particular must not only choose the optimal MR exam, interpret the images, and in some regulations, monitor the scan execution, but they must also preliminarily and carefully evaluate the patient’s clinical history to identify any contraindications or risks related to the MR examination and provide information to the patient on the relative benefits and risks [12].

From what can already be seen, with the existence of competing guidelines even at a very broad level, it can be deduced that the European MR safety landscape is heterogeneous, and different countries implement variations of the international safety guidelines, sometimes at a normative/legislative level, and sometimes as non-binding national recommendations [16, 17].

In Italy, for instance, a well-established framework for MR safety has been in force for decades. Current legislation [18] addresses patient, worker, and public safety with the same law. Two MRSEs apical roles are established in the decree, nominated as a trust-base assignment by the employer, one for the “clinical safety” and “diagnostic efficacy” of the MR system (a radiologist in 100% of cases) and one for physical aspects MR safety (a Medical Physicist in 82% of cases—2017 INAIL data) [19]. The two safety experts sign together acceptance tests and local safety rules, while the final responsibility of each medical exam lies with the radiologist on shift.

Austria has a similar legislative structure, where the figure of the safety officer is coded in the law [20]. The responsibilities and competencies, as well as the training to become an MRSO, were defined and certified in two ÖNORMS (Austrian official norms) part 1 and part 2 which were developed together with the Austrian Standards Institute. This certified education has significantly raised the awareness of MR safety in Austria and has put the expertise in MR safety and the implementation of MR safety measures and organizational procedures on a higher level with a transfer of know-how from well-trained MRSOs to colleagues in their institutions.

However, in other European countries, the clinical MR safety workflow is not generally present in the legislation, which mostly receives at a national level the IEC norms and ICNIRP guidelines for medical devices [21].

In this variegated context, we, as the ESR MR quality and safety working group, decided to conduct a survey among medical professionals to understand how, Europe-wide, the various institutions where MR is performed organize their internal workflows including: how the national and international guidelines on safety are incorporated into their routine, what kind of safety training the personnel has, how patient screening and scanning is performed, and whether accidents have occurred and were reported.

## Methods

An anonymous survey questionnaire was distributed between April and June 2023 through ESR member societies to healthcare professionals. The survey was distributed to the European Full and Allied Science Members in the ESR with the following professions: Medical Physicists, Radiographers/MR Technologists, and Radiologists. This totaled 27,475 members across 51 countries. Additionally, the survey was promoted through the ESR National Societies Committee Delegates, which included the presidents of all 47 National Institutional Members of the ESR, the Quality, Safety, and Standards Committee Delegates, encompassing National and Subspecialty Society Representatives, totaling 53 societies. All delegates and participants were encouraged to share the survey within their radiological communities, making it challenging to estimate the exact reach as some may have shared it outside their societies. Moreover, the survey was distributed through the EFRS, which, according to the website, has 100,000 members, the EFOMP which has 9100 declared members, and the ESMRMB which has fewer than 1000 members. While overlap is possible between the members of the societies, we estimate that the potential reach of the survey is between 100,000 and 150,000 individuals in at least 51 countries. It is important to note that this does not constitute a representative sample of the population of radiology professionals in Europe in the statistical sense, as no sampling strategy aimed at preventing bias was implemented [22].

The questions were divided into six broad categories:

1. Characteristics of the institutions.
2. Guidelines and awareness.
3. Internal organization and training.
4. Scanning practices.
5. Accidents.
6. Opinions on MR safety.

The actual survey questions are available in the appendix (Electronic Supplementary Material). While the survey was targeted to the countries affiliated with European societies, no specific restriction was posed in the definition of “European”. However, the respondent indicated the country where their institution was located. Results that were incomplete were only accepted if they contained more than the basic demographic information. The responses were manually checked for obvious outliers/mistakes (e.g., confounding the number of scanners present at a site with the number of monthly/yearly scans performed).

To compile a list of national guidelines, the responses to question 7 (“Provide a reference [to national guidelines on MR safety]”, see Appendix) were manually checked by one of the authors (F.S.) and, when necessary, translated into English using Google Translate (Google LLC, Mountain View, California, USA). They were included if they were considered to contain operative information about the safe clinical MR scanning of patients and were considered to have the nature of a national guideline or recommendation (e.g., by endorsement from a national society of radiology or medical professionals), in agreement among the authors of this manuscript.

If no valid response to question 7 was given following a positive response to question 6 (“In your country/region, as far as you know, are their national guidelines on MR safety?”), a generic search for guidelines was performed with the following modalities:

1. Performing a web search using Google search (Google LLC, Mountain View, California, USA) on the terms “MR safety” + [country name] (in English).
2. Performing the same search with the query translated into the official language (or the predominant official language in case of multiple official languages) of the country.
3. Performing an artificial-intelligence-assisted web search using ChatGPT (v4 with web search capability, OpenAI, San Francisco, California, USA) with the prompt “Find online the [country name] guidelines on MR safety. You have to search in [country language]”.

A similar search (replacing “guideline” with “law”) was performed to confirm the answer to question 8.

## Results

### Demographics

**Fig. 1:**
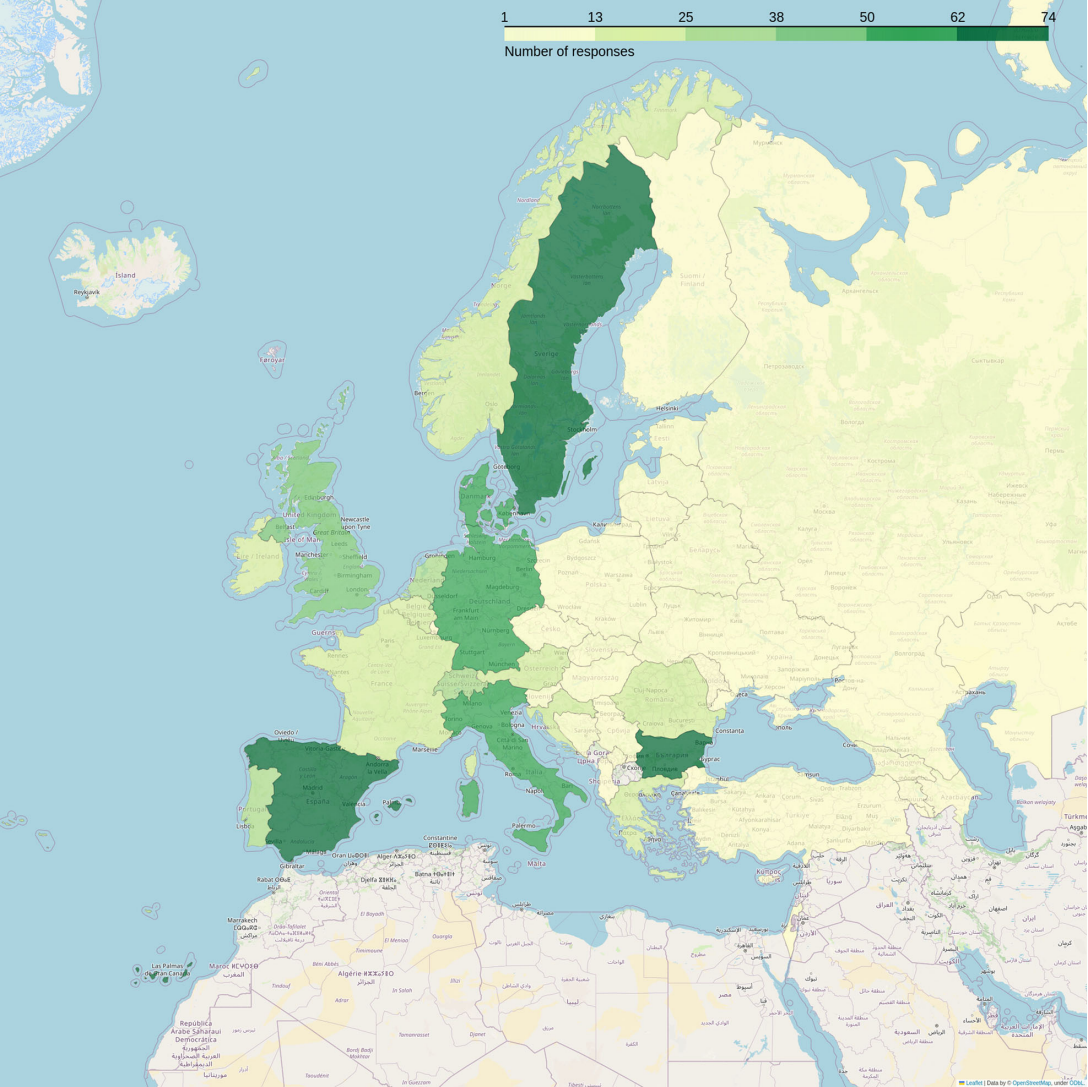
Number of valid responses received

793 responses were obtained from 44 different countries (Fig. 1), resulting in an estimated response rate of 0.5–0.8%, and a representation of 86% of the countries directly contacted by the ESR. Many respondents were radiologists (60%), followed by radiographers/MR technologists (28%), and physicists/engineers (10%). University/Research Hospitals comprised 61% of the responses, 31% were non-university/non-research hospitals, and 8% were outpatient facilities.

Many scanners were either 1.5-T (reported by 684 responders) or 3-T (506), with 78 respondents indicating possession of scanners below 1.5-T and 43 indicating scanners above 3-T.

### Guidelines and awareness

At least 50% of the respondents from the Czech Republic, UK, Italy, Sweden, Netherlands, France, Germany, and Austria, and a single respondent from Kosovo, reported that they were aware of the existence of national guidelines. Links to the national guidelines as indicated by respondents of the survey that could be confirmed are reported in Table 1. Not all links could be confirmed, as some did not refer to guidelines, but rather to articles on specific practices or generic overviews on MR safety. In contrast, at least 50% of the respondents from Albania, Italy, France, Austria, and Belarus reported that MR safety was mandated by law in their country. While this is definitely confirmable for Italy [18] and Austria [20], we were not able to confirm the existence of such laws for other countries. We cannot be certain whether this arises from our inability to exhaustively survey the local knowledge (although the effort was made to search the literature and the internet in local languages) or from false beliefs held by the respondents.

**Table 1:** Links to national safety guidelines, as reported by the respondents, that could be confirmed

Country	Link to guidelines
Austria	https://www.bdb.at/Service/NormenDetail?id=341737
Czech Republic	http://www.cesradiol.cz/dwnld/CesRad_1001_69_75.pdf
France	https://g4-hdf.fr/wp-content/uploads/2021/11/JFR2021-Recommandations_SFR_VF.pdf
Germany*	https://leitlinien.dgk.org/2017/mr-untersuchungen-bei-patienten-mit-herzschrittmachern-und-implantierbaren-kardioverter-defibrillatoren/
Ireland	https://www.hpra.ie/docs/default-source/Safety-Notices/sn201433_mri_safetynotice_060814.pdf?sfvrsn=2
Italy	https://www.gazzettaufficiale.it/eli/gu/2021/03/16/65/sg/pdf
The Netherlands	https://www.nvmbr.nl/publicatiebestanden/Leidraad%20veilig%20werken%20met%20MRI%20voor%20MBBers%20-%201911.pdf
Sweden	https://swedrad.se/mrsakerhet
Switzerland	https://www.ampec.ch/_static_files/SiMRI.pdf
Turkey	https://www.turkrad.org.tr/assets/DernektenHaberler-Pdf/MR-Guvenlik-Kilavuzu-09-12-2019.pdf
United Kingdom	https://www.gov.uk/government/publications/safety-guidelines-for-magnetic-resonance-imaging-equipment-in-clinical-use

The authors take no responsibility regarding whether these guidelines are officially endorsed by the respective professional societies. This list is based on self-reporting by the respondents of the survey and might not be complete

^*^ Guideline limited to cardiac devices

According to the answers to question 7, in which multiple respondents indicated the European directive receiving the ICNIRP guidelines [21] exposure of the general public and in professional settings are regulated by this framework, but no specific structure or responsibility for clinical MR scanning is generally mandated.

Overall, 37.8% of the respondents were aware of the existence of national guidelines, and 29.1% of the respondents were aware of the existence of a legal mandate for an MR safety structure.

### Internal organization and training

Of the respondents, 77.2% reported that their institution had a clear concept of the handling of MR safety in a clinical environment. The responsible person for MR safety was a radiologist in 44% of the cases, a radiographer in 32.4%, and a physicist/engineer in 15.8%. Of the remaining 7.8% who responded “Other”, the indicated responsibility was most commonly shared by a multidisciplinary task force or working group.

The proposed MRMD/MRSO/MRSE structure was only adopted by 38.6% of the respondents overall and was more widely adopted in Italy and Austria (where a clearly identified responsible person is expressly mandated by law).

In relation to specific MR safety-related training, more than half (52.3%) of the respondents reported that nobody in their institution had received any specific training. Of those that did, 27.9% never refreshed their training, the rest refreshed it at least once every 5 years, with 39.7% refreshing it at least once every two years, and 21.6% refreshing it every year. The training was mostly internally organized for all employment categories, with physicists/engineers receiving the highest share of external training (30.9%) and radiologists the lowest (17.6%).

**Fig. 2:**
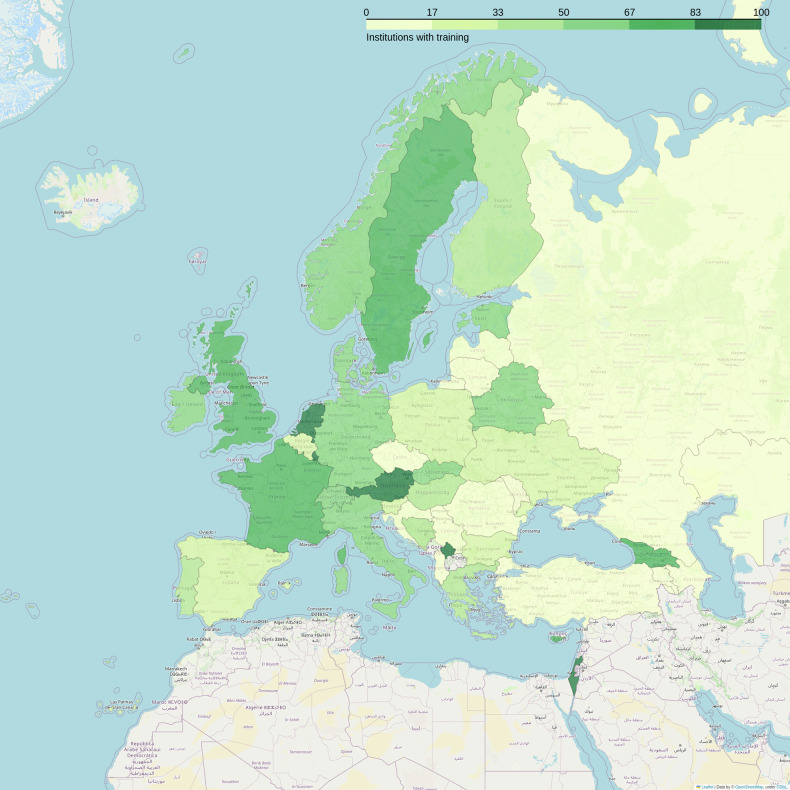
Percentage of institutions where any personnel with specific MR safety training is present

Geographically, in 15 countries (France, UK, Ireland, Italy, Switzerland, Austria, Germany, The Netherlands, Denmark, Norway, Sweden, Slovakia, Belarus, Kosovo, and Georgia; of note: Kosovo and Belarus, had 1 and 2 responses, respectively) at least 50% of the respondents reported that their institution employed personnel (radiologists, radiographers/MR technologists, and/or physicists/engineers) with specific MR safety training, either internally or externally organized (Fig. 2).

When internal training is organized, the teaching is performed by a single individual in 67.0% of the reported cases, by a team of 2 in 25.2% of the cases, and by a team of three or more in 7.7% of the cases. A physicist is involved (as sole teacher or part of the team) 50.7% of the time, a radiographer 45% of the time, a radiologist 36.4% of the time, and an external teacher 8.9% of the time.

The content of the training is not necessarily defined by the same individuals that teach the course, but the distribution of teamwork vs single individuals and of competencies involved is very similar, with the exception that the involvement percentages of radiographers and radiologists are roughly inverted, with a radiologist being involved 45.2% of the times, and a radiographer 38.8%.

### Scanning practices

**Fig. 3:**
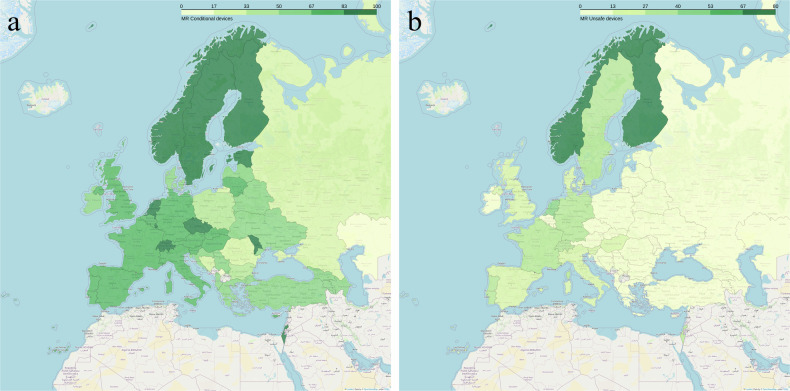
Percentage of institutions where MR-conditional (**a**) and MR-unsafe (**b**) cardiac devices are routinely scanned

As a representative example of “complex” scanning situations, we asked about the practice of scanning cardiac devices. MR-conditional cardiac devices are reported to be scanned routinely in 75% of the responses from university/research hospitals, in 68% of the responses from a non-university/non-research setting, but only in 42% of the responses from outpatient facilities. There is an observable divide between Western and Eastern Europe, with Eastern Europe generally scanning a smaller percentage of MR-conditional devices (Fig. 3a). MR-unsafe cardiac devices are reported to be scanned only in 27% of the responses from university/research hospitals, in 14% of the responses from outpatient facilities and in 10% of the responses from non-university/non-research contexts.

For both types of devices, a radiologist assumes the responsibility (either alone or in a team) in many reported cases (66.3% for MR-conditional devices, and 64% for MR-unsafe devices), whereas there is a responsible cardiologist in 35.8% of the MR-unsafe device scans, and in 24.4% of the MR-conditional device scans.

In the scanning routine, uncertainty regarding the MR compatibility of a patient was very relevant, with 75.3% of the respondents reporting that they refused at least one scan in the last 12 months because they were unsure about the MR safety evaluation, and 26.7% refused more than 5 scans in the last 12 months for the same reason.

### Accidents

Overall, 63% of the respondents declared that their institution has a well-defined incident reporting procedure in place. In Azerbaijan, Denmark, Estonia, Israel, Kazakhstan, Luxembourg, Malta, Moldova, Norway, United Kingdom, Netherlands, Ireland, Sweden, and Switzerland, at least 80% of the respondents reported the existence of an incidence reporting procedure (of note: Azerbaijan, Estonia, Kazakhstan, Luxemburg, and Moldova had fewer than three responses).

The number of patient injuries that occurred in the last 5 years was: 96 (12.1%) reported at least 2 projectile events with patient injuries (5 reporting at least 5 events), 100 (12.6%) reported at least 2 RF burns events (7 reporting at least 5 events), and 41 (5.2%) reported at least 2 implant-related injuries (4 reporting at least 5 events).

Personnel were also injured: 46 (5.8%) reported at least two cases of personnel injury due to projectiles, and 9 (1.1%) reported at least two cases of implant-related personnel injury.

Material damage due to projectiles was reported in 157 cases (19.8%, at least two events in the last 5 years).

Please note that the above figures refer to the number of responses received, which might not correspond to the actual number of accidents that occurred, as multiple respondents can be from the same institution and thus report the same accident.

### Opinions about MR safety

**Fig. 4:**
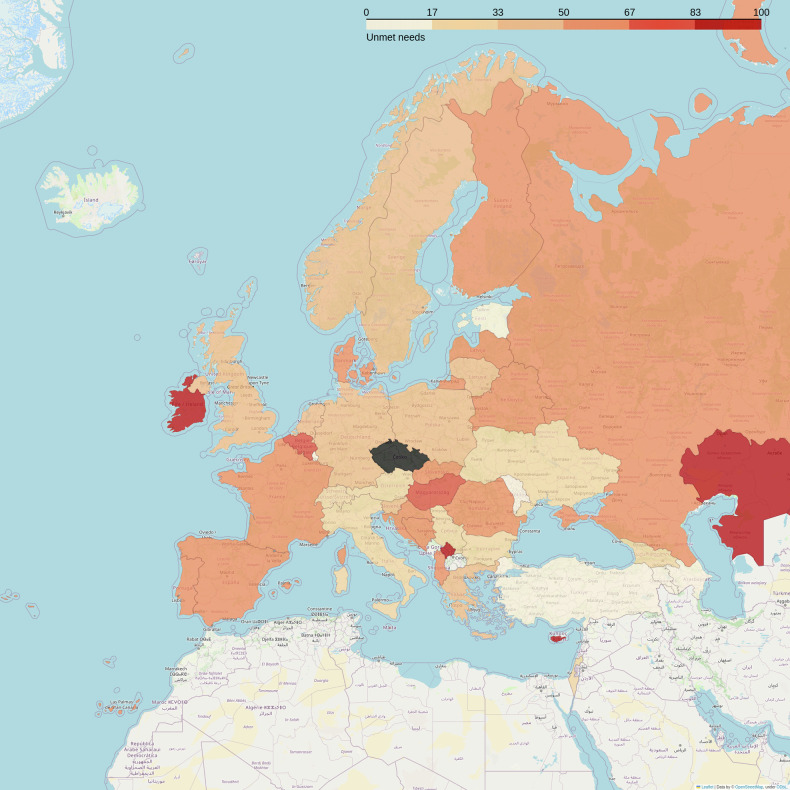
Percentage of responses that report unmet needs in terms of MR safety. The color black means “no answers received for this question”

The benefits of having an MR safety program were recognized by 91.4% of the respondents. Unmet needs were reported by 55.9% of the respondents (Fig. 4). In the free feedback area regarding unmet needs, the following were mentioned most often:

- Specialized training and refresher courses, standardized at a national or international level.
- Clear and updated guidelines.
- Training for non-MR personnel, including referring physicians.

It is important to notice that many responses specifically mentioned the need for continuous education/refresher courses.

## Discussion

This survey revealed a very heterogeneous European landscape in terms of MR safety. While the majority of institutions, at every level, have institutional guidelines to handle MR safety decisions and adverse events, coordination is lacking. This is probably not due to a lack of guidelines, as pointed out in the introduction, but rather to a lack of awareness of said guidelines which is related to the lack of coordinated education of personnel at every level.

This uncertainty leads to a potential under-providing for patients who could benefit from MR scans, as indicated by the low number of scans performed on patients with cardiac devices, and by the high number of scans refused due to uncertainty (based on the answers to question 24, “Have you ever refused to perform a scan because you were unsure about the MR safety assessment of the patient in the last 12 months?”).

However, the lack of scanning of patients with cardiac devices (assumed in our study to be representative of cases where safety management is complex) in outpatient facilities and smaller hospitals can also be explained by practical and economic considerations, due to the need for extra personnel and/or equipment, specifically, personnel able to reprogram the implanted device [23]. In fact, a cardiologist is often responsible for the scanning of cardiac devices everywhere in Europe.

Our survey specifically focused on cardiac devices, because they are widespread active implants, and there is now accepted evidence that even MR-unsafe devices can be safely scanned off-label [23]. We therefore considered this a good benchmark for the level of diagnostic offer of each facility.

In terms of clinical practice, the vast majority of institutions are currently using field strengths of 1.5 T and 3 T. However, it is to be expected that lower-field scanners will become more common, with various manufacturers starting to offer cheaper alternatives. While the lower-field scanners generally pose fewer safety concerns, it is important that specific, evidence-based procedures are established [24]. Specifically, it is important to remember that implants that are MR-conditional at a specific field strength, might not be safely scanned at lower fields than the ones specified.

Similarly, higher field strengths are also significantly present in Europe, and proper training for these field strengths should be made available to the relevant institutions.

The finding about the reported accidents, involving both patients and personnel, and resulting mainly from ferromagnetic attraction and RF-induced burns, is worthy of attention. More than one in ten respondents reported accidents resulting in an injury in the previous five years, which means that the MR risk of injury is non-negligible, and proper mitigation strategies, including thorough incident evaluation and reporting procedures, must be set in place in every institution. More thorough surveys of MR-related accidents performed in Sweden [5, 6] and Denmark [4] give a more detailed picture of the nature of such accidents.

It is however important to notice that the absolute number of respondents who reported an accident might not correspond to the number of accidents that effectively occurred, as we could not identify respondents from the same institution who might therefore have reported the same accidents more than once. At the same time, our sample is likely to be biased, as individuals who are more sensitive to MR safety concerns (potentially including those who have experienced an accident), are more likely to respond to the survey. On the other hand, underreporting of MR-related incidents is a reality [1, 4–6]. Another potential source of bias is the language of the survey, which was English and was not localized for different countries. We hypothesize that, for some countries, this could have led to a smaller number of overall responses and an underrepresentation of smaller facilities that do not employ English-speaking personnel. Similarly, for countries from which few responses were collected, we cannot rule out that they all originate from one or a few institutions only. A combination of the above reasons might have contributed to the final overrepresentation of university hospitals in our final sample, when they are in reality, a smaller fraction of all healthcare providers. Nevertheless, the good geographical spread and the high number of responses overall give reasonable confidence in our conclusions.

As a general conclusion, the picture of the MR safety landscape in Europe that emerges from this study is fragmented. While most institutions recognize the importance of institutional guidelines and procedures, specific training, and especially continuous education, is lacking, and we can assume that each institution delegates the management of MR safety to medical professionals with training in general radiology, but not with additional specific training in MR safety. There is significant geographical inhomogeneity in the implementation of specific training, which suggests that any effort towards standardizing MR safety practices in Europe should not happen at a continental level but rather at a national or, ideally, regional level. Interestingly, the regional differences seem to be more relevant than the differences in the nature of the facility in our sampled population, with similar levels of training in university and non-university hospitals and outpatient facilities.

Considering the current fragmentation and the need to harmonize the MR safety training for all professionals involved, we see the role of our ESR MR safety and quality working group, where all relevant societies are already represented, as the coordinator for the definition of a basic MR safety education curriculum. In contrast to some existing educational offers, we do not suggest that the primary goal of the training should be the formation of individuals with MRSO/MRMD/MRSE qualifications, as these are roles defined by local or internal guidelines and legislation, but rather the initial and continuous education of each person who has a relation to an MR facility.

To achieve this goal, we propose a general, four-tiered framework, with the expectation that every professional involved in patient management and decision-making reaches the learning outcomes of module 3.

**Fig. 5:**
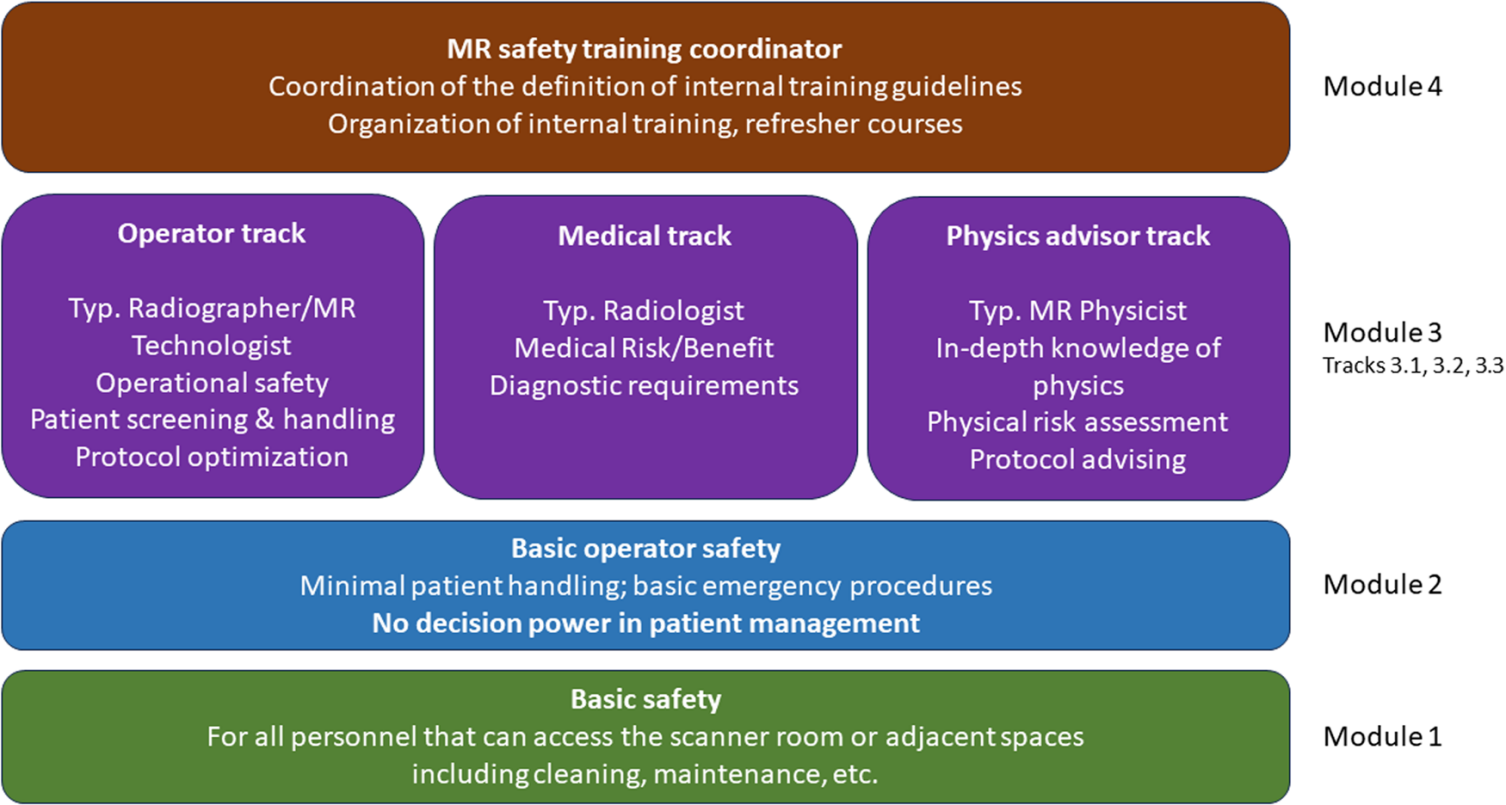
Proposed framework for the definition of a harmonized MR safety curriculum

A sketch of this framework is shown in Fig. 5. The first two common modules define the required training for all personnel operating in the vicinity of an MR scanner, and for those individuals involved in the handling of a patient in the MR room, but not in the scanning or decision process, respectively. In module 3, the framework gets divided into three tracks: the “operator” track, primarily fulfilled by radiographers/MR technologists, the “medical” track, primarily fulfilled by radiologists, and the “physics advisor” track, primarily fulfilled by MR physicists. The definition of the curriculum of each track will be defined with the primary input of the relevant European societies.

The fourth module, (MR safety training coordinator) is devoted to a person that could assume, in compatibility with local regulations, the role of coordinator for the definition of internal training guidelines and organization of internal training and refresher courses. We expect that only one or a few individuals per institution will require module 4.

The creation of such a curriculum will allow the capillary and decentralized diffusion of MR safety training that is required in such a diverse European scenario, and will allow for differences in implementation according to the local regulations. As our results show that internal training is prevalent and necessary in the European reality, this approach will leave each institution free to decide the modality in which they fulfill the objectives, provided that the required expertise is present. It will also allow different actors in the radiological education space to develop and adapt their own independent training programs in a harmonized fashion. The future steps of our working group will be to work with the representatives of the various professions to define and adapt the content of each track, ensuring that each individual will be trained in a way that respects the interdisciplinary approach required for effective MR safety management.

## Supplementary information

ELECTRONIC SUPPLEMENTARY MATERIAL

## Data Availability

The data generated during and/or analyzed are available from the institutional author upon reasonable request.

## Abbreviations

EFOMP: European Federation of Organizations for Medical Physics
EFRS: European Federation of Radiographer Societies
ESMRMB: European Society of Magnetic Resonance in Medicine and Biology
ESR: European Society of Radiology (ESR)
MR: Magnetic resonance
MRMD MR: Medical director
MRSO MR: Safety officer
