# Supplementary material for: The European MR safety landscape
Source: Insights Imaging. 2024 Oct 7;15:238. doi: 10.1186/s13244-024-01813-6 (PMC11458850; doi:10.1186/s13244-024-01813-6)
Supplement: Supplementary file 1 — ELECTRONIC SUPPLEMENTARY MATERIAL [file 13244_2024_1813_MOESM1_ESM.pdf]

The European MR Safety Landscape

ELECTRONIC SUPPLEMENTARY MATERIAL

Appendix: Survey questions

General

1. Where is your institution located?
2. Institution type
3. How many MR scanners do you have in your institution?
4. Field strength
5. What is your profession?

Safety

Guidelines

6. In your country/region, as far as you know, are there national guidelines on MR safety?
7. If yes, provide a reference (optional)
8. In your country/region, is a specific MR Safety concept/framework/program mandated by law?
9. Do you have an MR Safety concept/framework/program in your institution?
10. Who is primarily responsible for MR safety?
11. Do you follow the MR Medical Director/MR Safety Officer/MR Safety Expert (MRMD/MRSO/MRSE) structure of MR Safety responsibility?

Education

12. Do you have personnel with specific training/certification in MR Safety?
13. If yes, what percentage of each collaborator group has specific education on MR Safety?
    a. Radiologists
    b. Radiographers
    c. Physicists/Engineers
    d. Other staff members (e.g. anesthetists, nurses) (please specify which and what training)
14. What kind of training do they have?
    a. Radiologists
    b. Radiographers
    c. Medical/MR Physicists, Engineers
    d. Other staff members - Options
15. If you have an internal MR safety training program, who teaches it?
16. Who is responsible for the content of the program?
17. How often does your personnel undergo an MR safety refresher course?
18. Do you perform MR scans of **MR conditional** active cardiac devices (pacemakers, ICDs, …) at your institution?
    a. If yes, who is responsible for the evaluation/authorization?

19. Do you perform MR scans of **MR unsafe** active cardiac devices (pacemakers, ICDs, …) at your institution?
    a. Who is responsible for the evaluation/authorization?

Incidents and reporting

20. Do you have a clear procedure for MR incident reporting?
21. Have you had MR Safety-related incidents resulting in **patient injury** in the last 5 years?
    a. Projectile/Ferromagnetic attraction (How many?)
    b. RF burns (including tattoos) (How many?)
    c. Implant-related injury (How many?)
    d. Other
22. Have you had MR Safety-related incidents resulting in **personnel injury** in the last 5 years?
    a. Projectile/Ferromagnetic attraction (How many?)
    b. Implant-related injury (How many?)
    c. Other
23. Have you had MR Safety-related incidents resulting in **equipment damage** in the last 5 years?
    a. Projectile/Ferromagnetic attraction (How many?)
    b. Equipment malfunction (e.g. damage to electronics/mechanics) (How many?)
    c. Other
24. Have you ever refused to perform a scan because you were **unsure** about the MR Safety assessment of the patient in the last 12 months?

Final questions

25. Are there unmet needs in your institutions in terms of MR Safety?
    a. If yes, which?
26. Do you feel that an MR Safety program is/would be beneficial?
    a. How?
27. Based on your experience, do you have some further comments/ideas on how MR safety can be improved?
